# Simvastatin up-regulates adenosine deaminase and suppresses osteopontin expression in COPD patients through an IL-13-dependent mechanism

**DOI:** 10.1186/s12931-016-0424-6

**Published:** 2016-08-24

**Authors:** Kittipong Maneechotesuwan, Kanda Kasetsinsombat, Adisak Wongkajornsilp, Peter J. Barnes

**Affiliations:** 1 Division of Respiratory Diseases and Tuberculosis, Department of Medicine, Faculty of Medicine Siriraj Hospital, Mahidol University, 2 Prannok Road, Bangkok, 10700 Thailand; 2 Department of Pharmacology, Faculty of Medicine Siriraj Hospital, Mahidol University, Bangkok, Thailand; 3 Airway Section, National Heart and Lung Institute, Imperial College, London, UK

**Keywords:** Simvastatin, Adenosine deaminase, Osteopontin, IL-13, COPD

## Abstract

**Background:**

Adenosine deaminase (ADA) and osteopontin (OPN) may play opposing roles in the pathogenesis of COPD. Deficiency of ADA results in enhanced adenosine signaling which up-regulates OPN expression. Although statins suppress OPN in cancer cells, little is known about their effects on ADA and OPN in COPD patients.

**Methods:**

We extended a previous randomized double-blind placebo crossover study to investigate the effects of simvastatin (20 mg/day) on sputum ADA and OPN expression and explored the underlying signaling pathways involved by conducting in vitro experiments with cigarette smoke extract (CSE)-treated monocyte-derived macrophages (MDM) from COPD patients and healthy subjects.

**Results:**

Simvastatin decreased sputum IL-13, OPN and CD73, while increasing ADA expression, irrespective of inhaled corticosteroid treatment and smoking status in parallel to increased inosine levels. The degree of simvastatin-restored ADA activity was significantly correlated with the magnitude of changes in pre-bronchodilator FEV_1_. Mechanistic exploration showed that CSE enhanced the expression of IL-13, which induced an increase in OPN and inhibited ADA mRNA accumulation in MDM from COPD patients but not healthy subjects through a STAT6-dependent mechanism. Simvastatin treatment inhibited IL-13 transcription in a dose-dependent manner, and therefore diminished the IL-13-induced increase in OPN and restored IL-13-suppressed ADA. There was no effect of simvastatin on adenosine receptors in CSE-stimulated MDM, indicating that its effects were on the adenosine pathway.

**Conclusion:**

Simvastatin reversed IL-13-suppressed ADA activity that leads to the down-regulation of adenosine signaling and therefore inhibits OPN expression through the direct inhibition of IL-13-activated STAT6 pathway. Inhibition of IL-13 may reverse the imbalance between ADA and OPN in COPD and therefore may prevent COPD progression.

**Electronic supplementary material:**

The online version of this article (doi:10.1186/s12931-016-0424-6) contains supplementary material, which is available to authorized users.

## Background

Chronic obstructive pulmonary disease (COPD) is driven by chronic inflammation and tissue remodeling process in response to noxious particles or gases [1, 2]. Currently, there are no effective therapies that alter disease progression and improve survival [2] due to the complexity of signaling pathways that maintain chronic inflammation and tissue destruction [3].

Adenosine plays a key role in airway inflammation and remodeling in COPD [4]. Extracellular adenosine was produced upon cell damage to balance tissue repair against excessive airway remodeling in COPD [5–10]. Conversely, in prolonged or repeated tissue injury, chronic adenosine elevation can activate signaling pathways that promote tissue injury [5, 9]. Extracellular adenosine production is regulated by ecto-5'-nucleotidase (CD73); the rate-limiting step that converts AMP to adenosine [11]. CD73 expression and activity is markedly increased in patients with severe COPD, suggesting the high production of adenosine [3]. Adenosine can either interact with adenosine receptors or be transported into cytosol via facilitated nucleoside transporters [12]. Adenosine can be deaminated to inosine by adenosine deaminase (ADA) [13]. Adenosine is upregulated in IL-13-transgenic mice through the suppression of ADA activity and mRNA accumulation [14]. The synergistic effect of adenosine and IL-13 may contribute to the severity of airway inflammation and fibrosis in COPD [14]. Suppression of ADA activity together with CD73 up-regulation promotes adenosine production in the COPD lungs [3]. The association between defective ADA activity and COPD was demonstrated in ADA-deficient mice [15]. The pathological features of COPD was prevented and reversed by lowering adenosine levels with exogenous PEG-ADA [14–17].

Osteopontin (OPN) can function both as a matrix protein and a pleiotropic cytokine. OPN expression is regulated by IL-13 that can be induced by cigarette smoke extract [18, 19]. OPN accumulation in smokers correlates with the degree of airflow limitation [20]. Sputum OPN is significantly higher in COPD patients than in healthy smokers in agreement with the extent of emphysema [21]. In COPD lungs, OPN is primarily localized in alveolar macrophages and to a lesser extent in epithelial cells, T cells and fibroblasts [22]. Both adenosine and OPN are involved in COPD pathogenesis. ADA deficiency causes OPN-dependent neutrophilia and alveolar air-space enlargement [22]. Increasing adenosine signaling in severe COPD is directly associated with increased OPN transcripts [3, 22]. Therefore, the simultaneous inhibition of OPN as well as adenosine might be an additional strategy for prevention of COPD progression in particular deterioration of lung function caused by airway fibrosis.

Statins inhibit the synthesis of the cholesterol isoprenoid intermediates farnesylpyrophosphate (FPP) and geranylgeranyl pyro-phosphate (GGPP) [23]. Statins suppressed OPN mRNA and protein expression in an ovarian cancer cell line due to several diverse homologous cis-acting consensus sequences in human OPN promoters [24, 25]. In addition, this suppression may result from statin-depleted isoprenoids [26] that affects the expression of OPN [27]. However, the effect of simvastatin in the context of IL-13-regulated adenosine and OPN in COPD patients is not known.

We have demonstrated that simvastatin reversed IL-13-suppressed ADA activity in COPD patients that leads to the down-regulation of adenosine signaling and therefore inhibits OPN expression through the direct inhibition of IL-13-activated STAT6 pathway. Simvastatin-restored ADA activity positively correlated with the magnitude of changes in pre-bronchodilator FEV_1_. Inhibition of IL-13 may reverse the imbalance between ADA and OPN in COPD and therefore may prevent COPD progression.

## Methods

### Patients

Outpatients aged 45–80 years with a diagnosis of COPD, defined in accordance with the European Respiratory Society (ERS) consensus statement [28], who were current or ex-smokers with ≥10 pack-year history were recruited. They had a pre-bronchodilator forced expiratory volume in 1 s (FEV_1_) of < 80 % predicted normal values and a post-bronchodilator FEV_1_/forced vital capacity (FVC) < 70 %. Exclusion criteria have been described previously [29]. Patients who suffered acute exacerbations before study period were excluded. All subjects and donors gave written informed consent to a study protocol approved by the ethical committee of Faculty of Medicine Siriraj Hospital (Si323/2013).

### Study design

The study design has been described in detail previously [29]. The study was a 4-week randomized double-blind crossover study with a 4-week washout period comparing the effect of oral simvastatin treatment (20 mg once daily) with that of a matched placebo on sputum cytokine biomarkers and airway inflammation in COPD. During the 2-week run-in period and throughout the study, subjects continued their usual COPD medication and withdrew statin therapy for 4 weeks prior to the study entry if they were taking regular treatment. The study was listed on all appropriate clinical trial registries (ClinicalTrials.gov identifier: NCT01944176). Methods pertaining specifically to the current analysis will be described here.

### Measurement

Demographic measurements were recorded on the first clinical visit (visit 1 screening). Induced sputum and blood samples were collected before and after treatment periods for analysis of sputum cytokines. Spirometry was measured at all study visits. The description of sputum induction and processing of sputum samples, and other methodology related to this study can be found in Additional file 1.

### Statistical analysis

Data are presented as means ± SD or median (interquartile range; IQR) as appropriate. Response to simvastatin on sputum mediator levels versus placebo was assessed by general linear model for the standard 2 × 2 crossover design. When variables were unsuitable for this, the within-patient treatment differences were calculated and then analyzed by paired t-test for parametric and Wilcoxon Signed Rank test for non-parametric data. The effects of ICS treatment and smoking status on the response to simvastatin was analyzed by unpaired t-test. The r_s_ correlation coefficient was determined for the correlation of ADA and OPN with baseline pre-BD FEV_1_ and absolute changes in ADA and pre-bronchodilator (BD) FEV_1_ using Spearman’s rank correlation test and Pearson correlation coefficients, respectively. Statistical analysis for multiple comparisons was performed using one-way ANOVA and Welch test with Games-Howell correction for equal variances not assumed or Bonferroni corrections for equal variances assumed. Dunnett t tests was used for dose–response analysis. All statistical tests were two-sided, and significance was accepted at the level of 95 % and *P* < 0.05 using PASW statistics 18 (SPSS, IBM, Somers, NY).

## Results

**Fig. 1:**
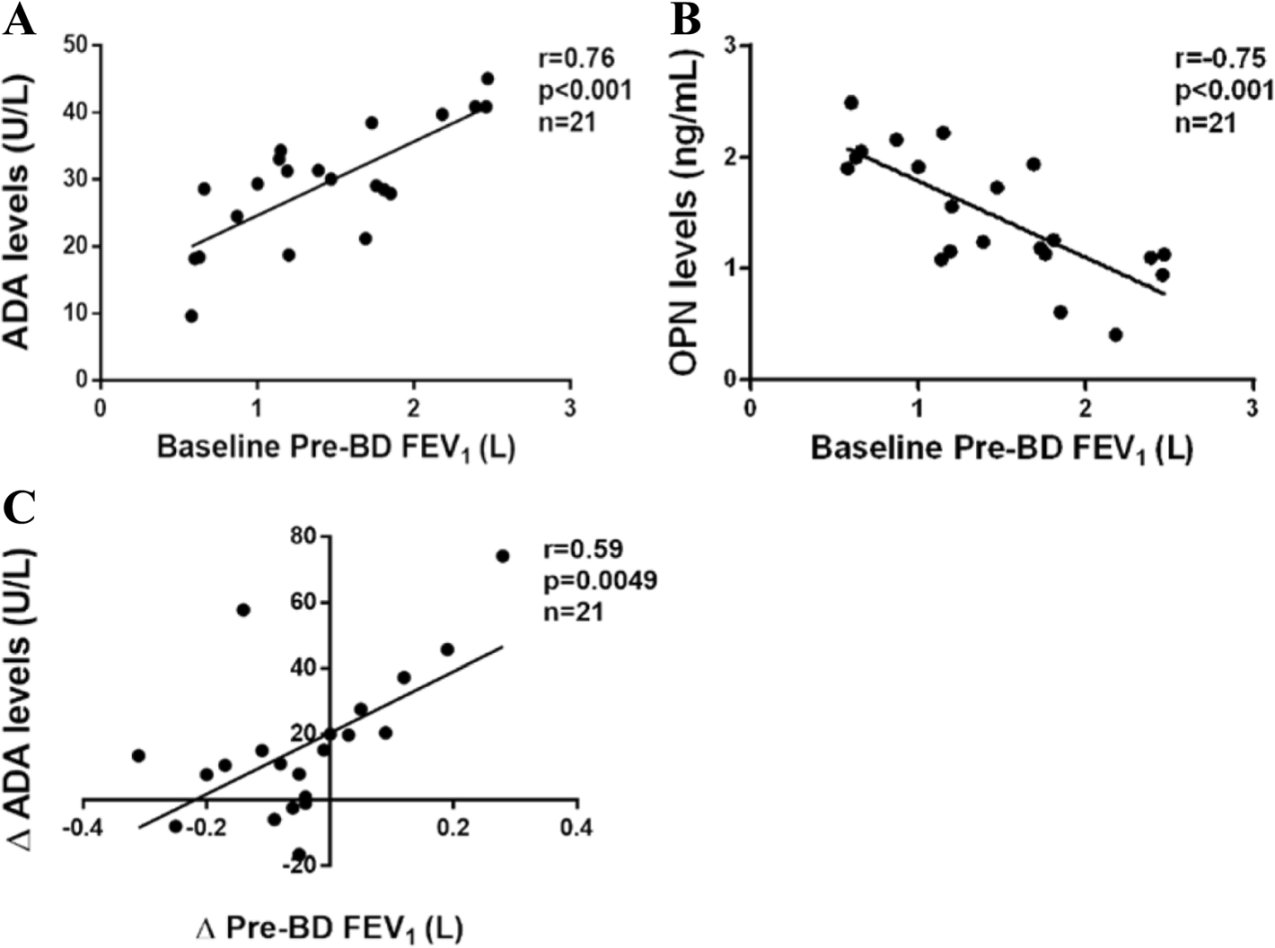
The data shows a positive correlation between baseline pre-BD FEV_1_ and ADA (**a**) and negative correlation with OPN levels (**b**) and the association between the magnitude of changes in ADA levels and the degree of pre-BD FEV_1_ improvement (**c**)

Flow of subjects through the study, demographic data, sputum cytology in response to simvastatin were previously described (Table 1) [29]. Pre-BD FEV_1_ values were positively correlated with baseline ADA but negatively correlated with OPN (r_s_ = 0.76; *p* < 0.001, r_s_ = −0.75; *p* < 0.001, respectively) (Fig. 1a, 1b).

**Table 1:** Patient characteristics

Clinical parameters	All patients (*n* = 21)
Age (yr)	68.4 ± 7.8
Sex: M: F	19:2
Smoking (pack-yr)	36.7 ± 28.9
Ex-smoker: current smoker (n)	16:5
Pre-BD FEV_1_/FVC (%)	53.9 ± 11.5
Post-BD FEV_1_/FVC (%)	55.7 ± 11.6
Post-BD FEV_1_ (L)	1.55 ± 0.6
Post-BD FVC (L)	2.7 ± 0.7
Δ FEV_1_ (mL)	115.2 ± 108.0
BD reversibility (%)	9.5 ± 8.9
DLCO (ml/mmHg/min)	57.4 ± 21.2
DLCO/VA (ml/mmHg/min/l)	62.4 ± 14.2
ICS/LABA use (n)	8
LAMA use (n)	1
Triple therapy (n)	7
Oral bronchodilator (n)	8
Previous statin therapy (n)	8

Data are presented as mean ± SD unless otherwise indicated

*BD* bronchodilator, Δ change in, *DLCO* diffuse lung capacity to carbon monoxide, *FEV*_*1*_ forced expiratory volume in 1 s, *FVC* forced vital capacity, *ICS* inhaled corticosteroid, *LABA* long-acting β_2_ agonist, *LAMA* long-acting muscarinic antagonist, *VA* alveolar volume

### Simvastatin enhanced ADA transcription and translation

Simvastatin increased ADA transcripts in sputum cells (*p* = 0.0002) and ADA levels in sputum supernatants (*p* < 0.001) (Table 2). The magnitude of the increase in ADA transcripts was 2.8 folds (95 % CI 1.3–4.2, *p* = 0.001) and ADA levels were 23.3 U/L (95 % CI 15.0–31.6, *p* < 0.001) (Table 3). Neither ADA transcription (*p* = 0.84) nor translation (*p* = 0.47) was altered in placebo-treated COPD. This effect was independent from the treatment sequence (simvastatin and placebo, *p* = 0.7 for ADA mRNA, *p* = 0.19 for ADA). ICS did not alter the effect of simvastatin on ADA (*p* = 0.73). Smoking status did not influence ADA response to simvastatin (*p* = 0.31). The ADA enhancement was associated with pre-BD FEV_1_ improvement (r =0.59; *p* = 0.0049) (Fig. 1c).

**Table 2:** Baseline induced sputum cytology and inflammatory marker levels and before-after each treatment

Variable	Baseline	Placebo		*P* value	Simvastatin		*P* value
		Before	After		Before	After	
Sputum mediators							
ADA mRNA (fold change) (*n* = 13)	1.03 ± 0.06	1.04 [1.0-1.05]	1.01 [0.7-1.4]	0.84	1.0 [1.0-1.0]	2.7 [2.3-2.9]	0.0002
ADA (U/L)	29.5 ± 8.9	29.8 ± 10.4	28.3 ± 9.4	0.47	28.7 ± 10.7	50.6 ± 23.7	<0.001
OPN mRNA (fold change) (*n* = 13)	1.0 ± 0.0	1.0 [1.0-1.0]	2.2 [0.8-4.2]	0.026	1.0 [1.0-1.0]	0.4 [0.2-0.6]	<0.001
OPN (ng/mL)	1.5 ± 0.6	1.6 ± 0.7	1.8 ± 0.9	0.25	1.7 ± 0.6	0.9 ± 0.5	<0.001
CD73 mRNA (fold change) (*n* = 13)	1.0 ± 0.02	1.0 [1.0-1.01]	1.05 [0.97-2.3]	0.065	1.0 [1.0-1.01]	0.8 [0.2-1.02]	0.057
Total CD73 (%)	12.4 ± 9.1	9.6 [3.5-12.6]	4.8 [1.5-13.6]	0.76	12.3 [5.0-25.1]	5.7 [2.5-9.2]	0.0071
Inosine (nmol)	0.57 ± 0.1	0.59 ± 0.1	0.52 ± 0.1	0.0034	0.5 ± 0.2	0.8 ± 0.1	<0.001
IL-13 (pg/ml)	26.3 ± 7.9	25.4 ± 8.9	23.4 ± 11.7	0.36	27.8 ± 6.6	10.9 ± 8.2	<0.001

Data was presented as mean ± SD or median [IQR] as appropriate and indicated

*ADA* adenosine deaminase, *IL* interleukin, *OPN* osteopontin

**Table 3:** The effect of simvastatin on sputum cell count and cytokines

Sputum cells	Placebo (*n* = 21)	Simvastatin (*n* = 21)	*P* value	Treatment difference, simvastatin minus placebo (95 % CI)	Effects by order *P* value
Δ sputum ADA mRNA (fold change) (*n* = 13)	0.3 ± 1.4	2.8 ± 3.1	0.001	2.8 (1.3 to 4.2)	0.7
Δ sputum ADA (U/L)	−1.5 ± 9.4	22.0 ± 18.4	<0.001	23.3 (15.0 to 31.6)	0.19
Δ sputum osteopontin mRNA (fold change) (*n* = 13)	1.6 ± 2.5	−0.6 ± 0.3	0.012	−2.4 (−4.1 to −0.6)	0.69
Δ sputum osteopontin (ng/mL)	0.15 ± 0.6	−0.71 ± 0.6	<0.001	−0.85 (− 1.2 to −0.5)	0.92
Δ sputum inosine (nmol)	−0.1 ± 0.1	0.3 ± 0.2	<0.001	0.37 (0.29 to 0.43)	0.054
Δ sputum CD73 mRNA (fold change) (*n* = 13)	0.7 ± 1.3	−0.2 ± 0.6	0.01	−0.91 (−0.37 to −1.5)	0.99
Δ sputum CD73 (%)	0.58 ± 8.7	−7.5 ± 13.6	0.006	−8.3 (−13.8 to −2.7)	0.92
Δ sputum IL-13 (pg/mL)	−2.0 ± 9.9	−16.9 ± 8.3	<0.001	−15.0 (−20.4 to −9.6)	0.69

Data are presented as mean ± SD

*ADA* adenosine deaminase, *IL* interleukin, *OPN* osteopontin

### Simvastatin suppressed CD73 transcription and protein expression

Simvastatin decreased CD73 transcript and the number of CD73-expressing sputum cells (−0.91 folds (95 % CI −0.37 to −1.5), *p* = 0.01; −8.3 % (95 % CI −13.8 to −2.7), *p* = 0.006, respectively) (Table 3). This effect was independent from the treatment sequence (simvastatin and placebo, *p* = 0.99 for CD73 mRNA and *p* = 0.92 for CD73).

### Simvastatin treatment increased sputum inosine levels

Simvastatin significantly increased whereas placebo decreased sputum inosine (*p* < 0.001 and *p* = 0.0034, respectively) (Table 2). In addition, the magnitude of the increase in inosine levels was 0.37 nmol (95 % CI 0.29–0.43, *p* < 0.001) (Table 3).

### Simvastatin inhibited OPN transcription and translation

Simvastatin markedly decreased OPN transcripts in sputum cells (*p* < 0.001) and OPN levels in sputum supernatants (*p* < 0.001), whereas placebo treatment caused increased OPN transcription (*p* = 0.026) but not OPN levels (*p* = 0.25) (Table 2). The magnitude of the reduction in OPN transcripts was 2.4 folds (95 % CI −4.1 to −0.6, *p* = 0.012) and OPN levels were 0.85 ng/ml (95 % –1.2 to −0.5), *p* < 0.001) (Table 3). This suppression was independent from the treatment sequence (simvastatin and placebo, *p* = 0.69 for OPN mRNA and *p* = 0.92 for OPN). ICS did not alter the effect of simvastatin on OPN (*p* = 0.84). Smoking status did not influence OPN response to simvastatin (*p* = 0.73).

Because IL-13 is induced by adenosine and induces OPN expression [18, 30, 31], we investigated whether simvastatin suppresses IL-13 production in the airways of COPD patients. We found that simvastatin markedly decreased sputum IL-13 levels (−15.0 pg/ml (95 % CI −20.4 to −9.6), *p* < 0.001) compared with placebo (Table 3). However, we could not exclude the possibility that the effect of simvastatin on IL-13 was due to either the direct suppression or the consequence of decreased adenosine as a result of ADA augmentation or both as adenosine could stimulate IL-13 [30]. This prompted mechanistic studies in MDM from COPD patients.

### Mechanistic investigations in CSE-treated MDM

**Fig. 2:**
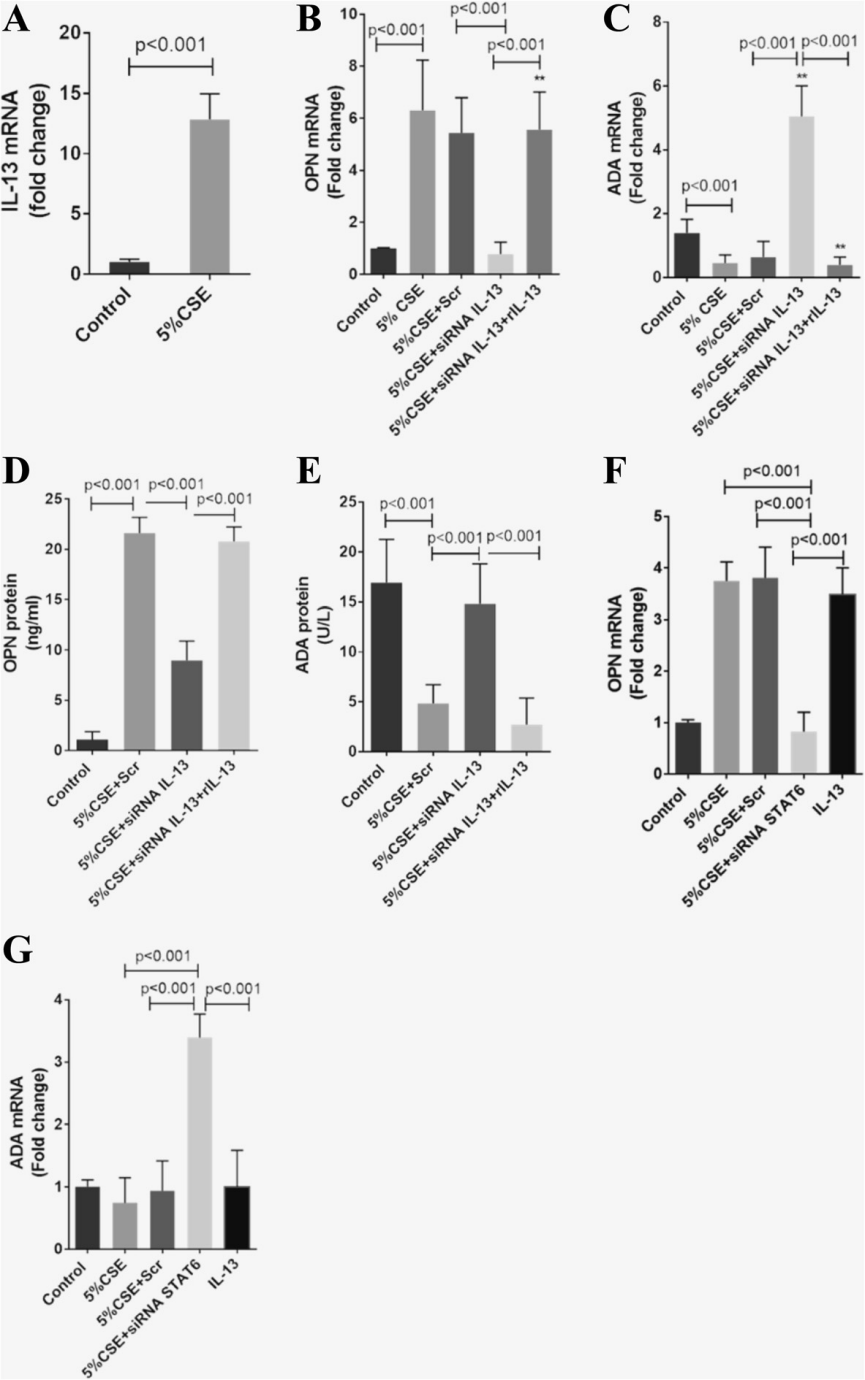
**a** The effect of CSE on IL-13 transcription. The effect of knockdown of IL-13 transcripts on OPN (**b**) and ADA mRNA expression (**c**), OPN (**d**) and ADA (**e**). The effect of knockdown of STAT6 on OPN (**f**) and ADA mRNA expression (**g**). Results are expressed as the means (±SD) values of six independent experiments from 6 COPD patients. **, *p* < 0.001 vs control

**Fig. 3:**
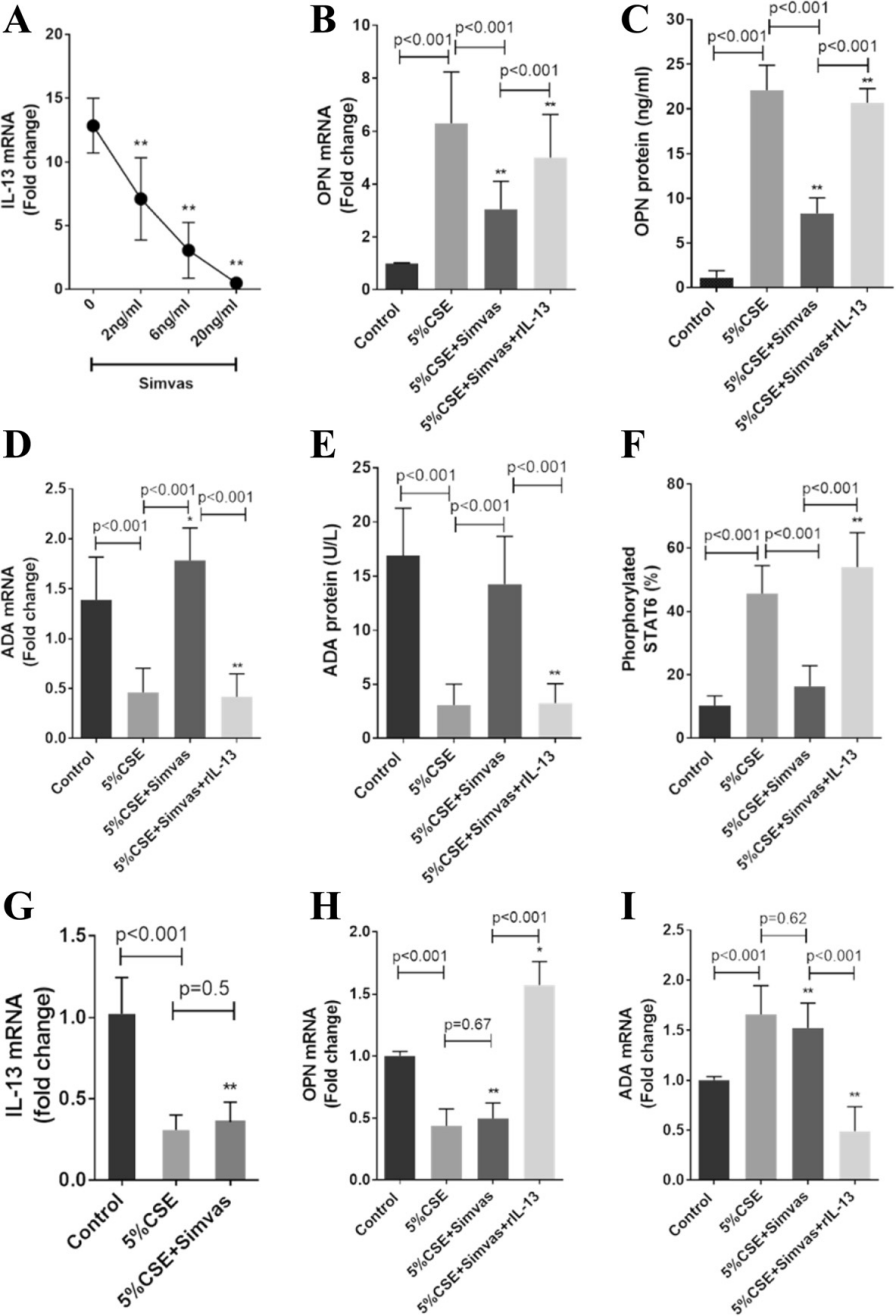
**a** Inhibitory effects of simvastatin on IL-13 mRNA expression in CSE-treated MDM from COPD patients in a dose-dependent manner. The inhibitory effects of simvastatin (2 ng/ml) on OPN mRNA expression (**b**) and protein concentration (**c**), and STAT6 phosphorylation (**f**). The enhancing effects of simvastatin (2 ng/ml) on ADA mRNA expression (**d**) and protein concentration (**e**). MDM from healthy subjects were used to demonstrate the effect of CSE and simvastatin on IL-13, OPN, and ADA transcription (**g**, **h**, **i**, respectively). This also illustrates the reversal of the above effects of simvastatin by the addition of exogenous human recombinant IL-13. Results are expressed as the means (±SD) values of six independent experiments from 6 COPD patients and 4 healthy subjects. *, *p* < 0.05 and **, *p* < 0.001 vs control

To determine whether IL-13 regulated OPN and ADA expression in CSE-stimulated MDM from COPD patients, we inhibited IL-13 using siRNA. CSE enhanced OPN and inhibited ADA transcription through IL-13 induction (mean fold change ± SD: 6.3 ± 1.9 vs 1.0 ± 0.02, *p* < 0.001; 0.46 ± 0.2 vs 1.39 ± 0.4, *p* < 0.001; 12.8 ± 2.1 vs 1.02 ± 0.2, *p* < 0.001, respectively) (Fig. 2a-c) whereas IL-13 mRNA expression was suppressed in CSE-treated MDM from normal subjects, resulting in downregulation of OPN and upregulation of ADA transcription (0.31 ± 0.1 vs 1.02 ± 0.2, *p* < 0.001; 0.44 ± 0.1 vs 1.0 ± 0.04, *p* < 0.001; 1.7 ± 0.3 vs 1.0 ± 0.03, *p* < 0.001, respectively) (Fig. 3g-i). IL-13 knockdown decreased OPN and increased ADA in both transcript and protein levels (mean fold change ± SD: 0.77 ± 0.4 vs 5.4 ± 1.3, *p* < 0.001; 9.0 ± 1.9 ng/ml vs 21.6 ± 1.6 ng/ml, *p* < 0.001 for OPN, 5.0 ± 0.9 vs 0.63 ± 0.5, *p* < 0.001; 14.8 ± 4.0 U/L vs 4.8 ± 1.9 U/L, *p* < 0.001 for ADA). These alterations could be completely reversed by exogenous IL-13 in both MDM from COPD patients (Fig. 2b-e) and healthy subjects (Fig. 3h and 3i), indicating the OPN positive regulation and ADA negative regulation by IL-13. The IL-13 effect was mediated through STAT6 as STAT6-knockdown antagonized CSE-induced OPN transcription induction and ADA transcription inhibition (mean fold change ± SD: 0.83 ± 0.38 vs 3.75 ± 0.37, *p* < 0.001; 3.4 ± 0.38 vs 0.75 ± 0.4, *p* < 0.001, respectively). Stimulation of STAT6 signaling with IL-13 induced OPN and inhibited ADA transcripts when compared with STAT6 knockdown (3.5 ± 0.52, *p* < 0.001 for OPN; 1.01 ± 0.58, *p* < 0.001 for ADA) (Figs. 2f and 3g).

We investigated whether simvastatin affected IL-13-mediated alterations to OPN and ADA expression in CSE-treated MDM from COPD patients. Simvastatin inhibited IL-13 mRNA expression in a dose-dependent manner (Fig. 3a). Simvastatin at therapeutic range (2 ng/mL) [32] was used in subsequent experiments. Simvastatin markedly decreased OPN transcripts and protein levels (mean fold changes ± SD, 3.0 ± 1.1 vs 6.3 ± 1.9, *p* < 0.001; 8.3 ± 1.7 ng/ml vs 22.1 ± 2.8 ng/ml, *p* < 0.001, respectively) (Fig. 3b, 3c) in MDM from COPD patients whereas simvastatin had no any effect on IL-13, OPN, and ADA in CSE-treated MDM from healthy subjects (0.36 ± 0.1 vs 0.31 ± 0.1, *p* = 0.5; 0.5 ± 0.1 vs 0.44 ± 0.1, *p* = 0.67; 1.5 ± 0.2 vs 1.7 ± 0.3, *p* = 0.62) (3g-i). This suppression in MDM from COPD patients was reversed when IL-13 was added (for OPN transcripts 5.0 ± 1.6 vs 3.0 ± 1.1, *p* < 0.001; for OPN levels 20.7 ± 1.6 ng/ml vs 8.3 ± 1.7 ng/ml, *p* < 0.001). In contrast, simvastatin markedly enhanced ADA transcripts and protein levels (1.8 ± 0.3 vs 0.46 ± 0.2, *p* < 0.001; 14.3 ± 4.4 U/L vs 3.1 ± 1.9 U/L, *p* < 0.001) that could be reversed by exogenous IL-13 (0.42 ± 0.2 vs 1.8 ± 0.3, *p* < 0.001; 3.3 ± 1.8 U/L vs 14.3 ± 4.4 U/L, *p* < 0.001) (Fig. 3d and 3e). Therefore, simvastatin inhibited IL-13 and consequently reduced OPN and increased ADA in CSE-treated MDM.

**Fig. 4:**
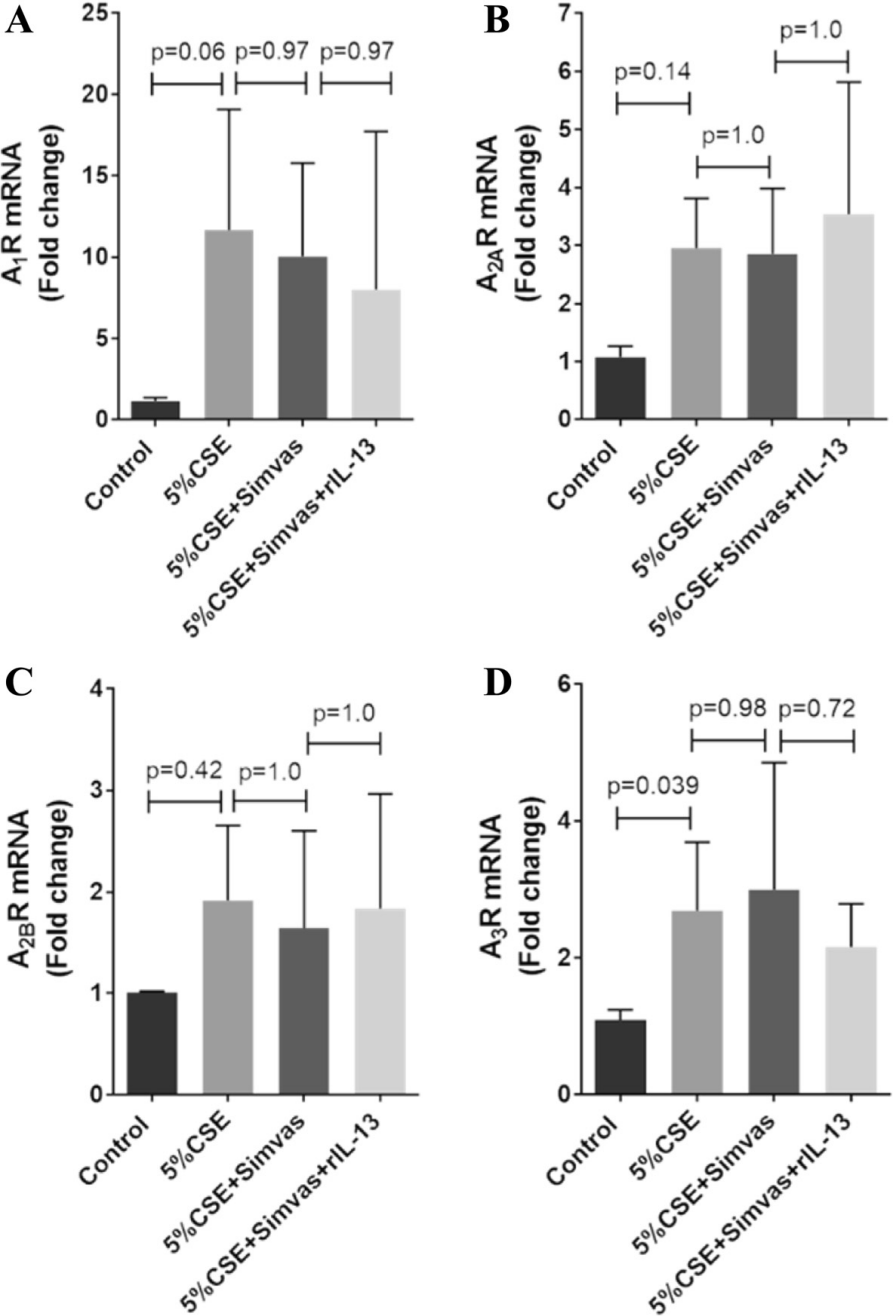
None of the effects of simvastatin on A_1_R (**a**), A_2A_R (**b**), A_2B_R (**c**) and A_3_R (**d**). Results are expressed as the means (±SD) values of six independent experiments from 6 COPD patients

We tested whether the simvastatin induced OPN suppression was mediated through inhibition of STAT6-dependent IL-13 signaling. CSE markedly increased, whereas simvastatin dramatically decreased STAT6 phosphorylation in MDM (45.7 ± 8.8 % vs 10.3 ± 3.0 %, *p* < 0.001; 16.4 ± 6.5 % vs 45.7 ± 8.8 %, *p* < 0.001, respectively) (Fig. 3f). The inhibition of STAT6 phosphorylation by simvastatin could be reversed by IL-13 (54.0 ± 10.7 %, *p* < 0.001). Simvastatin did not alter the expression of A_1_R, A_2A_R, A_2B_R or A_3_R, but CSE increased A3R expression (Fig. 4).

## Discussion

We have demonstrated for the first time that simvastatin enhances ADA expression while inhibiting osteopontin via inhibition of STAT6-dependent IL-13 signaling. In addition to ADA enhancement, simvastatin suppressed CD73 expression, both of which resulted in increasing sputum inosine. Our study confirmed the diametrically opposed role of OPN and ADA in patients with COPD. Osteopontin were inversely correlated, whereas ADA levels were directly correlated with pre-BD FEV_1_. Simvastatin-restored ADA activity was associated with improving pre-BD FEV_1_. Our mechanistic exploration in vitro suggested that CSE enhanced IL-13 that induced OPN but suppressed ADA in COPD-derived MDM via the STAT6-dependent pathway whereas in healthy-derived MDM, CSE suppressed IL-13, resulting in the reverse effect to OPN and ADA transcription. Simvastatin inhibited CSE-enhanced IL-13 in a dose-dependent manner and subsequently decreased STAT6 phosphorylation, leading to the reciprocal induction of ADA and inhibition of OPN. In addition, simvastatin did not affect adenosine receptor expression and had no inhibitory effects on IL-13, OPN, and ADA in CSE-treated MDM from healthy subjects.

Statins have strong immune-modulating effects in both the systemic and pulmonary inflammation, which could have substantial benefits in patients with COPD [33]. Anti-inflammatory effects of statins are mediated through inhibition of key inflammatory and remodeling pathways [33]. This is supported by the recent study showing that statins significantly reduced airway and pulmonary neutrophils in stable COPD [34]. Our previous study also demonstrated that statin therapy could suppress IL-17 and restore IL-10 in COPD although this was associated with reduction in alveolar macrophages but not neutrophils as previously explained [29]. Therefore, statins may attenuate progressive decline in lung function although there have been very few studies investigating signaling molecules simultaneously involved in both inflammatory and fibrogenic activities including OPN and IL-13.

IL-13 polymorphism is associated with an increased risk of COPD [35–37]. Alveolar or extrapulmonary macrophages produce IL-13 in response to rhinovirus or parasitic infection in mice [38, 39]. CSE may induce IL-13 producing murine macrophage M2 polarization in mice, either through its direct effect on JAK/STAT3 or indirectly through mast cell-associated mechanisms [40, 41]. In addition, a previous study showed that RSV infection enhanced cigarette smoke-induced IL-13 gene expression, but not IL-13 release, in mice although the cellular source of IL-13 was not identified [42]. CSE induces IL-13 secretion by macrophages in mice. However, there are very few data addressing the ability of CSE to induce an IL-13 response in human macrophages from patients with COPD. We have demonstrated that CSE markedly induced IL-13 response with STAT6 activation in MDM from COPD patients. However, this was not the case for the effect of CSE on IL-13 in healthy-derived MDM that revealed in reverse, possibly preventing healthy MDM from the establishment of IL-13/OPN axis. Therefore, CSE-stimulated COPD-derived MDM could be used in our study to investigate the effects of simvastatin on CSE-induced IL-13-mediated OPN and ADA expression.

OPN induces airway neutrophilic inflammation and airway remodeling [22] and its expression is increased in patients with COPD [3, 21]. Simvastatin may suppress pro-inflammatory and induce anti-inflammatory cytokines in COPD [29]. Furthermore, simvastatin inhibits IL-13 inducible OPN gene in airway epithelial cells. However, whether IL-13 regulates OPN expression and whether simvastatin affected this in patients with COPD is unknown. Therefore, the mechanistic understanding of how OPN accumulation is induced by IL-13 and how simvastatin mediates its inhibitory effects on IL-13-induced OPN accumulation in COPD patients may provide a new therapeutic target in future. The present study is the first to investigate whether CSE induces OPN transcription in an IL-13-dependent manner and showed that silencing of IL-13 or STAT6 with siRNA resulted in the down-regulation of CSE-enhanced induction of OPN and the up-regulation of CSE-suppressed expression of ADA. Furthermore, our findings confirmed and extended the previous data that in addition to inhibition of CSE–induced increase in OPN expression [18, 27], simvastatin also inhibited CSE-induced increase in IL-13 production by MDM from patients with COPD in a dose-dependent manner. In addition, the effect of simvastatin on CSE-induced OPN expression could be overcome by the addition of exogenous IL-13, confirming that inhibition of CSE-induced OPN by simvastatin was mediated through IL-13 suppression. Simvastatin reduces IL-13 levels with varying doses in a mouse asthma and bleomycin-induced pulmonary fibrosis model [43, 44]. However, our result is in contradiction to previous studies in Th2-biased animal models in that simvastatin induced Ym1 in dendritic cells which resulted in the augmentation of IL-13 release from Th2 cells [45]. This leads us to speculate that there are varying effects of simvastatin on IL-13 which are dependent upon specific cell types and types of inflammation.

Several studies have shown that IL-13 is strongly induced by adenosine in ADA knockout mice during an inflammatory and remodeling response [14, 30, 31]. During this response, IL-13 causes a progressive accumulation of adenosine and inhibits ADA activity and mRNA accumulation [14]. Our study demonstrates the inhibitory effect of IL-13 on ADA in patients with COPD, and that simvastatin treatment reverses IL-13-suppressed ADA in CSE-treated MDM from COPD patients. Restoration of ADA as well as inhibition of CD73 in the airways of COPD patients by simvastatin could be the mutually beneficial conversion of adenosine to inosine as exemplified by an increase in sputum inosine levels. However, we could not exclude the possibility that the reversal of ADA suppression may diminish the IL-13-induced increase in adenosine [14] and the possibility that this may be enhanced by simvastatin-inhibited IL-13 in our COPD patients.

IL-13 and adenosine stimulate each another in an amplification pathway that may contribute to progression and/or chronicity of IL-13, which at least in part is augmented by increased adenosine receptor expession [14]. A_1_R, A_2B_R and A_3_R are elevated in the lungs of IPF models. Previous data suggested that there was elevated expression of A_2B_R in macrophages in COPD lung specimens [3]. Persistent activation of A_2B_R signaling maintains inflammatory mediators, such as IL-6, IL-8, IL-13 and OPN [3], [46–48]. In contrast, another study demonstrated elevation of A_2A_R and A_3_R but down-regulation of A_2B_R transcripts [49]. The discrepancy between both studies may be related to differences in disease severity and smoking status. The present study has demonstrated that simvastatin failed to suppress adenosine receptors. This results indicates that the inhibitory effects of simvastatin on IL-13 and OPN response is mediated through down-regulation of adenosine signaling resulting from modulation of adenosine rather than its receptors.

Our results were in the agreement with other previous studies showing the beneficial effects of statins [50–52] but differed from the results of the recent study (STATCOPE) demonstrating no benefit [53]. A plausible explanation for the discrepancy between STATCOPE and other previous studies including our study is the inclusion of a large percentage of COPD patients with coexisting overt cardiovascular disease who are ‘statin users’ and ‘non-users’, but would benefit from statin therapy. These comorbid phenotypes of COPD are likely to express a poor prognosis due to one or a combination of undertreated pulmonary inflammation, unrecognized systemic inflammation or subclinical cardiovascular disease. These comorbid conditions are strongly associated with an increased risk of hospitalization with acute exacerbations and greater mortality [54–56]. Therefore, there is possibility that many patients with COPD who have not been prescribed with statins in other previous studies do badly from undertreatment, a hypothesis mentioned by STATCOPE investigators to explain this discordance [53]. Another explanation for the STATCOPE findings is that COPD patients with coexisting clinical and subclinical cardiovascular disease underlying acute exacerbations has been all but excluded [57, 58].

The present study has demonstrated the involvement of IL-13 in CSE-induced OPN and simultaneously suppressed ADA in MDM and the consistent imbalance between IL-13-regulated OPN and ADA in patients with COPD. The balance toward IL-13-induced OPN can be reversed by simvastatin treatment. However, whether the reversal would provide the long-term benefit in lung function decline for patients with COPD required further study.

## Conclusions

This study provides the better understanding of the molecular mechanisms underlying the effects of simvastatin on ADA/OPN balance in COPD patients. Simvastatin treatment induces ADA and inhibits OPN expression in the airways and monocyte-derived macrophages from COPD patients. This effect is mediated through the direct inhbition of IL-13 activated STAT6 pathway which drives the imbalance between ADA and OPN towards the latter.

## Additional file

Additional file 1: Simvastatin up-regulates adenosine deaminase and suppresses osteopontin expression in COPD patients through an IL-13-dependent mechanism. (DOCX 21 kb)

## Abbreviations

ADA: Adenosine deaminase
CSE: Cigarette smoke extract
IL: Interleukin
MDM: Monocyte derived macrophage
OPN: Osteopontin
