# Supplementary material for: Simvastatin up-regulates adenosine deaminase and suppresses osteopontin expression in COPD patients through an IL-13-dependent mechanism
Source: Respir Res. 2016 Aug 24;17(1):104. doi: 10.1186/s12931-016-0424-6 (PMC4997725; doi:10.1186/s12931-016-0424-6)
Supplement: Additional file 1: — Simvastatin up-regulates adenosine deaminase and suppresses osteopontin expression in COPD patients through an IL-13-dependent mechanism. (DOCX 21 kb) [file 12931_2016_424_MOESM1_ESM.docx]

**Simvastatin up-regulates adenosine deaminase and suppresses osteopontin expression in COPD patients through an IL-13-dependent mechanism: *Online Supplement***

Kittipong Maneechotesuwan

Division of Respiratory Disease and Tuberculosis,

Department of Medicine, Faculty of Medicine Siriraj Hospital,

Mahidol University, Bangkok, Thailand

Kittipong.man@mahidol.ac.th

Kanda Kasetsinsombat

Department of Pharmacology, Faculty of Medicine Siriraj Hospital,

Mahidol University, Bangkok, Thailand

kanda.kae@mahidol.ac.th

Adisak Wongkajornsilp

Department of Pharmacology, Faculty of Medicine Siriraj Hospital,

Mahidol University, Bangkok, Thailand

adisak.won@mahidol.ac.th

Peter J Barnes

Airway Disease Section, National Heart and Lung Institute,

Imperial College, London, UK

p.j.barnes@imperial.ac.uk

**SUPPLEMENTAL METHODS**

**Sputum induction and processing**

Sputum induction was performed as previously described [E1]. The supernatants were stored at –70°C until further analysis. For immunocytochemistry, cytospins were fixed with 4% paraformaldehyde (BDH Ltd., Poole, UK) and stored at –20°C. Total cell counts were recorded with on a hemocytometer, using Kimura staining. Cell viability was determined by Trypan blue exclusion before cytospins were undertaken. The slides were stained with May-Grunwald-Giemsa stain and differential cell counts were made by a blinded observer. Four hundred inflammatory cells were counted on two slides for each sample in a blinded manner. Differential cell counts are expressed as the percentages of total inflammatory cells. Samples with cell viability of greater than 70% and less than 30% squamous cell contamination were considered adequate for analysis.

**Preparations of cigarette smoke extract**

Cigarette smoke extract (CSE) was prepared using two full-strength Marlboro cigarettes with filters removed (Phillip Morris, VA), which were combusted through a modified 60-ml syringe apparatus into 20 ml RPMI 1640 as described **[**E2**].**

**Culture of monocyte-derived macrophages**

Whole blood was collected from COPD patients from Siriraj Hospital (Bangkok, Thailand) and from healthy volunteer subjects. This study was approved by the Institutional Review Board of the Siriraj Hospital **(**Si025/2007**)**, and all subjects gave written informed consent. Peripheral blood mononuclear cells were isolated by Isoprep (Robbins Scientific, Sunnyvale, CA) and resuspended in RPMI1640 medium supplemented with 2 mM L-glutamine 10,000 U/mL penicillin, 10 mg/mL streptomycin and 10% (v/v) fetal bovine serum (Invitrogen, Paisley, UK) and seeded into 12-well tissue culture plates at a density of 1x10^6^ cells/well. Monocytes were isolated from the PBMC fraction by adherence on to cell culture plates for 1 hour. After this time, the non-adherent cells were removed by aspiration and media replaced with the buffer above. Cells were cultured for 10 days prior to the induction of differentiation into macrophages with GM-CSF (750 IU/ml, Xiamen Amoy Top Biotech Co, China) and then further incubated until day 14 before acquisition. For the experiments, MDM were incubated with 5% CSE for 24 hours and further untreated or treated with simvastatin (Sigma) at different doses in the presence or absence of recombinant human IL-13 cytokine (10 ng/ml, Bio Legend, CA) for 24 hours.

**Knockdown of IL-13 and STAT6 expression**

RNAi was used to suppress IL-13 and STAT6 in MDM. Two ds 21-mer RNA oligonucleotides directed against IL-13 and STAT6 were synthesized by Qiagen (Manchester, UK). The target sequences for IL-13 are CAG CTC TCA GCC AAC GAG TAA, AAC AAG TTG TTT CAT TGA CTA, TTC ATT GAC TAT CAA ACT GAA and CAG CAT GGT ATG GAG CAT CAA. The target sequence for STAT6 is ACG GAT AGG CAG GAA CAT ACA. Cells were transfected with siRNA using DOTAP Liposomal Transfection Reagent (Roche Applied Science, USA), as described by the manufacturer. IL-13 protein expression was monitored by flow cytometry after 48 hours.

#### RNA isolation and Real-time qRT-PCR analysis

Total RNA was extracted from whole sputum cell pellets by using an RNeasy Mini kit (Qiagen, Valencia, CA), according to the manufacturer’s instructions. Total RNA was reverse transcribed into cDNA by using Improm-II Reverse Transcription system (Promega, Madison, WI). Real-time RT-PCR was performed with FastStart Universal SYBR Green Master (Roche, Mannheim, Germany) with an ABI PRISM 7900 thermal cycler (Applied Biosystems, Warrington, UK), according to the manufacturer’s instructions. Primers were purchased from First BASE Laboratory (Singapore): osteopontin sense: AGG AGG AGG CAG AGC ACA, antisense: CTG GTA TGG CAC AGG TGA TG; ADA sense: GGC TCG GCC GAA GTA GTA AA, antisense: TTA TAA AGG GCC TGG TCT TCC A; CD73 sense: TAT CCG GTC GCC CAT TGA, antisense: GTG CCT CCA AAG GGC AAT AC; IL-13 sense: GAG TGT GTT TGT CAC CGT TG, antisense: TAC TCG TTG GTC GAG AGC TG; A_1_R sense: TCT GCT CTG AGA CGG ATG GA, antisense: CCT CTG GCC AGT CTC TCC TAC A; A_2A_R sense: ATG GAG TTT GCC CCT TCC TAA, antisense: TCC CAA CGT GAC TGG TCA AG; A_2B_R sense: TCC TCT GGG TCC TTG CCT TT, antisense: CCA GGG TTC TGT GCA GTT GTT; A_3_R sense: TCC TCT TGG CCC ATC TCA CT, antisense: CTC TGC CTG CAG CTT TTT GG.

**Fluorescence-activated cell sorting (FACS) analysis**

Flow cytometric analysis was performed as previously described with modification [E3]. Briefly, MDM were washed with PBS containing 1% BSA (Sigma-Aldrich) and centrifuged at 300g. The cells were resuspended in PBS containing 1% BSA (FACS buffer) at 1x10^6^ cells/ml, fixed and permeabilized with FACSPerm for 20 min, and washed twice. For each test, cell suspension (50 μl) was incubated with antibody (2 μl) for 45 minutes at 4^o^C in the dark. Cells were then washed, and resuspended in FACS buffer and subsequently analyzed using a FACSort (Becton Dickinson, San Jose, CA). Up to 30,000 total events were collected per sample. To determine the expression of phosphorylated STAT6, cells were stained with phycoerythrin (PE) conjugated anti-human phospho-STAT6 (e-Bioscience, San Diego, CA). The respective negative controls were matched isotype controls. Data were analyzed using WinMDI version 2.9.

**Alkaline phosphatase immunostaining**

Indirect staining of sputum cells was performed using the alkaline phosphatase-anti-alkaline phosphatase (APAAP) method using a commercial kit (Vectastain Laboratories, Burlingame, CA) [E4]. Cells were permeabilized for 10 min with 0.5% Nonidet P-40 (NP-40, Sigma Chemicals, St Louis, MO) and blocked in 20% normal swine nonimmune serum (Vector Laboratories, Burlingame, CA) for 30 min at room temperature. Polyclonal anti-rabbit antibody was used to detect CD73 (Abcam, Cambridge, MA). After incubating with the secondary biotinylated goat anti-rabbit antibody, the immunoreaction was detected using the APAAP system to produce red staining. Slides were counterstained with hematoxylin for cellular identification, and examined under light microscopy. Anti-CD73 positive cells, which were identified by red immunoreactive signals on sputum cytospins, were counted by an experienced observer blinded to the clinical characteristics of the subjects. The immunopositive and immunonegative cells were counted at least 400 cells and expressed as a percentage.

**ELISA**

For detection of human OPN and IL-13 was performed according to the manufacturer’s instructions (Abcam, Cambridge, MA). The sensitivity was 50 pg/ml for OPN and 0.15 pg/ml for IL-13. Cytokine output was normalized to the concentration of protein.

Because sputum supernatant specimens contained 0.05% of DTT at the final concentration, we measured OPN, ADA and IL-13 in the control buffers in the absence and presence of DTT in order to determine whether it had potential effects on ELISA assay. We found that DTT at the final concentration 0.05% used for sputum processing did not affect OPN, ADA and IL-13 assays.

**ADA and inosine quantification assays**

Adenosine deaminase was determined by adenosine test kit (Diazyme Laboratories, Poway, CA) while inosine was quantitatively measured by fluorometric assay kit (Abcam, Cambridge, MA). The sensitivity was 0.1 nmol for inosine and the assay range for ADA was 0-200 U/L. The assays were performed according to the manufacturer’s instructions.

**REFERENCES FOR SUPPLEMENT**

E1 Maneechotesuwan K, Kasetsinsombat K, Wongkajornsilp A, Barnes PJ. Decreased

indoleamine 2,3-dioxygenase activity and IL-10/IL-17A ratio in patients with COPD.

*Thorax.* 2013;68(4):330-337.

E2 Walters MJ, Paul-Clark MJ, McMaster SK, Ito K, Adcock IM, Mitchell JA. Cigarette

smoke activates human monocytes by an oxidant-AP-1 signaling pathway: implications

for steroid resistance. *Mol Pharmacol*. 2005;68(5):1343-1353.

E3 Maneechotesuwan K1, Kasetsinsombat K, Wamanuttajinda V, Wongkajornsilp A,

Barnes PJ. Statins enhance the effects of corticosteroids on the balance between

regulatory T cells and Th17 cells. *Clin Exp Allergy*. 2013;43(2):212-22.

E4 Maneechotesuwan K, Ekjiratrakul W, Kasetsinsombat K, Wongkajornsilp A, Barnes PJ.

Statins enhance the anti-inflammatory effects of inhaled corticosteroids in asthmatic

patients through increased induction of indoleamine 2, 3-dioxygenase. *J Allergy Clin*

*Immunol*. 2010;126(4):754-762 e751.
